# Propranolol therapy of infantile hemangiomas: efficacy, adverse effects, and recurrence

**DOI:** 10.1007/s00383-013-3283-y

**Published:** 2013-03-22

**Authors:** Qiang Xiao, Qin Li, Bin Zhang, Wenlin Yu

**Affiliations:** Department of Plastic Surgery, General Hospital of Guangzhou Military Command (Liuhuaqiao Hospital), 111 Liu Hua Road, Yue Xiu District, 510010 Guangzhou, People’s Republic of China

**Keywords:** Propranolol, Infantile hemangiomas, Efficacy, Adverse effects, Recurrence

## Abstract

**Objective:**

To evaluate the efficacy, adverse effects, and recurrence of oral propranolol for treatment of infantile hemangioma.

**Methods:**

Participants were treated with oral propranolol three times daily, with inpatient monitoring of adverse effects. The starting dosage was 2 mg/kg per day, which had been for the remaining duration of treatment. Therapy duration was planned for 4–6 months; if there was significant relapse, the period of treatment was extended. A photograph based severity scoring assessment was performed by three observers to evaluate efficacy by visual analog scale (VAS).

**Results:**

Sixty-one infants [median age 3.3 (1.2–8.1) months] were included in the study. The median follow-up-time was 15 (6–20) months and 53 patients completed treatment at a median age of 10.3 (8.4–18.1) months, after a duration of 8.5 (4.5–14) months. In all patients, there was significant fading of color [with a VAS of −9 (−6 to −9) after 6 months] and significant decrease in size of the infantile hemangiomas [with a VAS of −8 (−3 to −10) after 6 months]. We did not observe any life-threatening adverse effects. The therapy was interrupted due to temporary aggravation of pre-existing bronchial asthma in one child. Four cases presented partial recurrences.

**Conclusions:**

Oral propranolol 2 mg/kg per day was a well-tolerated and effective treatment, mild adverse effects, and low recurrence for infantile hemangiomas. Propranolol should now be used as a first-line treatment in hemangiomas when intervention is required. Also, prospective studies should be needed in determining the most effective treatment dosage, optimum treatment duration, and exact mechanism of action of propranolol in future.

## Introduction

Infantile hemangiomas (IHs) are benign vascular tumors found in approximately 5–10 % of all newborns and infants up to 1 year. Although most do not need treatment due to their self-limited, approximately 10 % require medical intervention, include those involving the periorbital area, central face, airway, skin folds, anogenital area, sites at high risk for ulceration, dysfunction, or disfigurement. Systemic corticosteroids have been the mainstay of treatment for more severe IH; however, the response is variable, and side-effects are insidious and difficult to monitor. Moreover, corticosteroids work best at stopping further growth, but actual shrinkage only occurs in approximately one-third of patients [1]. Other treatment options for problematic IH include intra-lesional corticosteroids, vincristine, interferonalpha, cyclophosphamide, laser, surgery, or a combination of these therapies. However, each of these modalities has limited therapeutic benefit with its own side-effect profile and potential serious risks [2, 16].

Léauté-Labrèze et al. [3] reported the incidental finding that hemangiomas regress in children treated with propranolol, a non-selective beta-blocker used in treating infants with cardiac and renal conditions. This report has been met with great interest and enthusiasm and physicians all over the world have started to use propranolol for the treatment of problematic IH. Although a definite reduction or even disappearance of IH is well-documented with propranolol, the experience of many authors indicates that a number of IH recur after propranolol withdrawal, as has been stated in several articles, although the rate and the possible causes of recurrence have not been established [4–9]. The small number of cases treated worldwide, the lack of long-term protocols, the heterogeneity in the behavior of IH, and the short period of time that propranolol has been used for the treatment of IH make it difficult to assess main data associated with IH recurrence after therapy withdrawal [10–14].

In the present study, we report one of the largest case series to date, that of 64 infants treated with propranolol, including efficacy, side effects, and recurrence.

## Patients and methods

Propranolol was administered to 64 children with IH associated with life-threatening potential, functional risk, local complications, or cosmetic disfigurement. A treatment guideline was designed that was based on the known side effects of propranolol and in collaboration with pediatric cardiologists, hematologists, dermatologists, and plastic surgeons. After initial evaluation and informed consent and explanation of alternative therapeutic options, patients were scheduled for 3-day inpatient hospitalization. Before treatment was started, an electrocardiogram was performed to detect any pre-existing cardiac conduction disturbance. Serial photographs of the IH were obtained to evaluate the efficacy of propranolol. The dosage was 2 mg/kg per day. Parents were instructed to interrupt the administration of the drug if the child had a serious cough with dyspnea. Follow-up visits were scheduled monthly and at any time in case of complications. The dosage of propranolol was adjusted to the weight of the patient only in case of relapse of the hemangioma or a stop in regression after an initially observed decrease. At each clinic visit, blood pressure, heart rate, and blood glucose was measured, the effect of the treatment were determined, and possible adverse events were documented. We obtained serial clinical images to assess the rate and degree of recurrence. A visual scale to assess the severity of the IH was used. Briefly, three of the authors independently evaluated clinical images taken at baseline and in the follow-up visits. The observer documented changes in color and size of the lesions on a visual analog scale (VAS) ranging from −10 to +10 by comparing follow-up images to the baseline photograph pre treatment. On the VAS 0 represented the baseline photograph (pre-treatment), a decrease in color or size resulted in a − number, an increase in color or size in a + number.

### Statistical analysis

Data collected from the patients’ charts, ultrasound examination, and evaluation of photographs by VAS were entered into a computerized database. Median and range were calculated for continuos values. The Wilcoxon test was used to compare two related samples and the null hypothesis was rejected with a two-sided *p* value of <0.05.

## Results

**Fig. 1:**
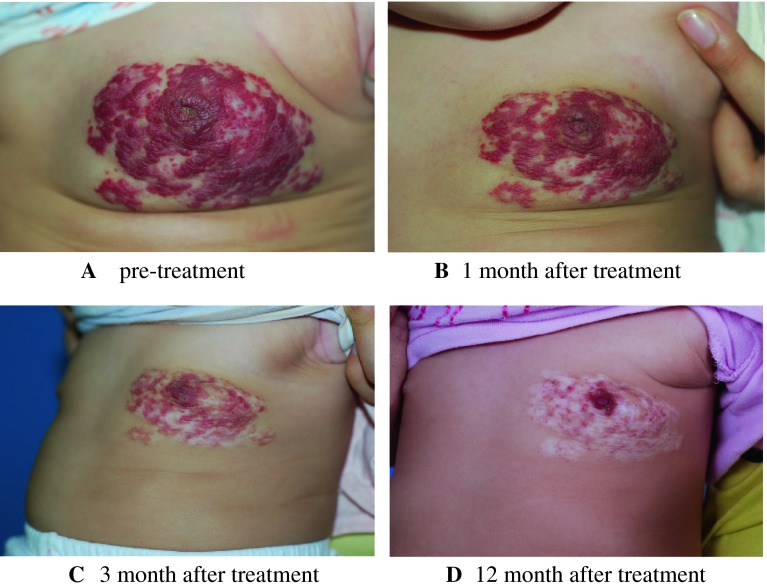
**a** Child present at 2 months of age before treatment. **b** Spontaneous regression after 1 month of propranolol treatment at 2 mg/kg per day. **c** Further improvement after 2 months. **d** Residual telangiectases at 12 months of age, after cessation of propranolol treatment

**Fig. 2:**
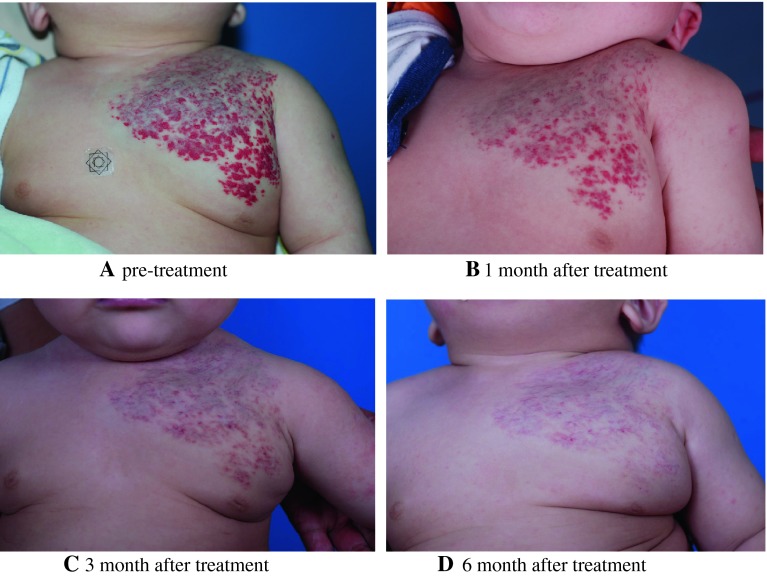
**a** Patient was referred aged 3 months with rapidly growing hemangioma in left chest. **b** After 1 month of propranolol treatment at 2 mg/kg per day. **c** After 3 months of propranolol treatment at 2 mg/kg per day. **d** After 6 months of propranolol treatment at 2 mg/kg per day

**Fig. 3:**
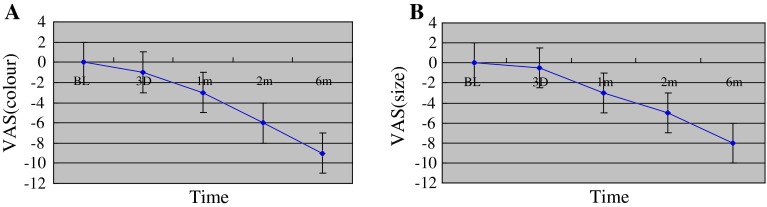
Changes of VAS regarding color (**a**) and size (**b**) of the hemangiomas during follow-up and treatment with propranolol (*BL* baseline, *d* days, *m* months); *p* < 0.01 for both parameters

Sixty-four patients with IH were included in the study. The relevant epidemiologic and clinical characteristics of the patients and details about the individual treatment indication and duration are shown in Table 1. The female-to-male ratio was 3.9:1. The median age at start of treatment with propranolol was 3.6 (0.5–9.1) months. Three patients were previously preterm infants born at 36 weeks of gestation and at initiation of treatment they were all older than 40 weeks of gestation. In two infants, laser therapy had been performed earlier without success and their IH continued to increase in size. All patients were treated with propranolol at a dose of 2 mg/kg per day. Fifty-three patients completed treatment at median age of 10.3 (8.4–18.1) months and after a median treatment duration of 8.5 (4.5–14) months. Eleven patients had ongoing therapy with propranolol at the time of writing this paper (Table 1). The median follow-up-time of all patients was 15 (6–20) months. Treatment response all IH stopped growing faded in color and became smaller (Figs. 1, 2). VAS measurements of photographic documentation were available at time points 0 (baseline), 3 days, 1, 2, and 6 months. Figure 3a, b demonstrates the changes of VAS values for color and size of the lesions over time. There was significant fading of color and decrease in size of the IH during the follow-up period compared to the photographs at baseline. Of note, the VAS already decreased significantly within the first 3 days of therapy for both, color and size. After 6 months, fading of color was reached with a VAS of −9 (−6 to −9) and shrinking of the lesions with a VAS of −8 (−3 to −10).

**Table 1:** Summary of baseline characteristics and treatment of infantile hemangiomas

Patient characteristics and treatment	*n* = 64
Female-to-male ratio	51:13
Type of hemangioma	
Superficial	25
Deep	22
Mix	17
Location of hemangioma	12
Head	7
Nose	15
Mouth	21
Periocular	8
Parotid area	5
Trunk	5
Limbs	6
Ulcerated hemangiomas	5
Age initiation of propranolol (months) median (range)	3.6 (0.5–9.1)
Duration of propranolol treatment (months), *n* = 64 median (range)	8.5 (4.5–14)
Age at end of propranolol treatment (months), *n* = 53 median (range)	10.3 (8.4–18.1)
Duration of propranolol treatment until stopped, *n* = 53 median (range)	8.5 (4.5–14)

### Side effects

No severe adverse events were noted in our patients. Thirteen (23.6 %) showed a reaction possibly due to the medication. Therapy was interrupted in one child who had temporary aggravation of preexisting asthma. All other observed adverse effects were mild and were tolerated without discontinuing the medication (Table 2).

**Table 2:** Complications and adverse effects

Observed adverse effects	*n* (%)	Propranolol terminated because adverse effects, *n*
Hypoglycemia	0	0
Hypotension	0	0
Bradycardia	1	0
Seizure	0	0
Restless sleep	3	0
Cold extremities	2	0
Gastrointestinal problems	6	0
Diarrhea,	4	
Constipation	2	
Bronchial asthma	1	1

### Recurrence

**Fig. 4:**
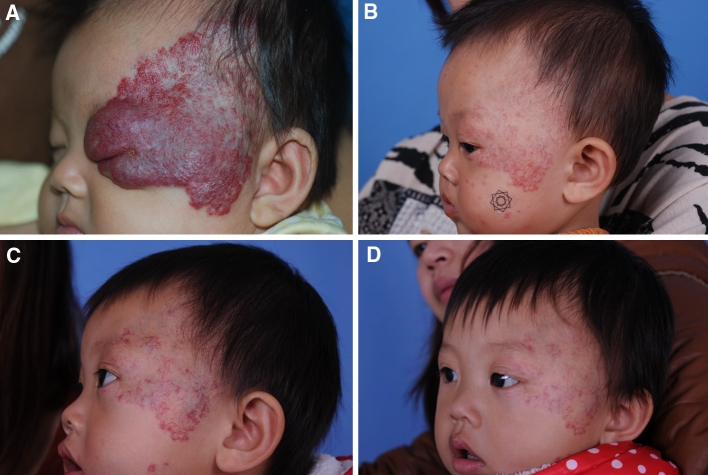
Clinical images of patient 1, **a** before starting treatment with propranolol; **b** at withdrawal; **c** recurrence at the age of 14 months; **d** clinical images after second treatment with propranolol

A summary of all cases is presented in Table 3 and Fig. 4.

**Table 3:** Visual scale scoring as evaluated three independent observers

Case location		Mean ± standard deviation			Recurring component	Segmental of focal	Retreatment
		Age before propranolol months	Age at propranolol termination months	Age at maximal recurrence months			
1	Left temple and periorbital	10 ± 2	1.7 ± 10	7.43 ± 16	Deep	Segmental	Yes, good response
2	Left chest	10 ± 1.5	1.7 ± 11	8.52 ± 15	Superficial and deep	Focal	Yes, good response
3	Upper lip	9 ± 3	3.54 ± 9	6.32 ± 15	Deep	Segmental	Yes, good response
4	Right arm	9 ± 7	0 ± 13	5 ± 21	Deep	Focal	No

## Discussion

Propranolol was the first clinically useful beta-adrenergic receptor antagonist. Invented by Sir James W. Black, it revolutionized the medical management of angina pectoris and is considered to be one of the most important contributions to clinical medicine and pharmacology of the twentieth century [15]. Beta-blockers may also be referred to as beta-adrenergic blocking agents, beta-adrenergic antagonists, or beta-antagonists. Propranolol is a non-selective beta-blocker. The levorotatory isomer of propranolol binds reversibly with β1- and β2-adrenoreceptors; both receptors have membrane stabilizing activity [16].

### Efficacy

The response of infantile hemangiomas to propranolol reported in the New England Journal of Medicine by Léauté -Labrèze et al. [3] catapulted the use of this treatment to first-line status among physicians managing this disease [17, 18]. Regulators of hemangioma growth and involution are poorly understood. Infantile hemangiomas are composed of a complex mixture of clonal endothelial cells associated with pericytes, dendritic cells, and mast cells. Hemangiomas usually appear a few weeks after birth and grow more rapidly than the infant does. This proliferative phase of hemangiomas is characterized histologically by plump endothelial cells with frequent mitosis, an increased number of mast cells and multilaminated basement membranes. Two major proangiogenetic factors are involved: basic fibroblast growth factor (bFGF) and vascular endothelial growth factor (VEGF). This period is followed by spontaneous slow involution, which shows apoptosis, and is morphologically characterized by flat, inactive, and normal-appearing endothelial cells in a matrix of the so-called "fibrous-fatty tissue" [19, 20]. In all patients, there was significant fading of color [with a VAS of −9 (−6 to −9) after 6 months] and significant decrease in size of the IH [with a VAS of −8 (−3 to −10) after 6 months]. Potential explanations for the therapeutic effect of propranolol on hemangiomas include vasoconstriction, which is immediately visible as a change in color, associated with a palpable tissue softening. Other included suggestions are a down-regulation of angiogenetic factors such as VEGF and bFGF and an up-regulation of apoptosis of capillary endothelial cells [3, 21]. There are also data published which indicate a selective role of propranolol in inhibiting the expression of MMP-9 (angiogenic and extracellular matrix degrading enzyme) and HBMEC (human brain microvascular endothelial cells). These facts may potentially add to propranolol’s anti-angiogenetic properties. HBMEC play an essential role as structural and functional components in tumor angiogenesis [22].

### Adverse effects

We did not observe any severe adverse effects, although there have been a few reports of serious adverse effects, particularly hypoglycemia. Adverse effects of beta-blockers for other medical indications have also been reported, including hypotension, bradycardia, bronchospasm, and hypoglycemia [23, 24]. Non-selective beta-blockers are competitive antagonists of catecholamines at the β-1 and β-2 adrenergic receptors. Beta-2 receptor blockade may result in hypoglycemia as a result of decreased glycogenolysis, gluconeogenesis, and lipolysis. Lawley et al. [10] reported the cases of two patients who received propranolol in the recommended dosage of 2 mg/kg per day. One experienced severe hypotension and the other severe hypoglycemia. Other authors have reported similar cases [6, 13, 17]. Burns et al. [25] stated that hypoglycemia can be associated with poor neurological outcome. Symptomatic hypoglycemia can be a serious complication of propranolol treatment. A propranolol dosage of over 4 mg/kg per day seems to put the pediatric patient at risk for development of hypoglycemic events [26]. Propranolol had to be discontinued in one patient because of bronchial hyperreactivity during viral infections. Bronchial hyperreactivity is a direct effect of non-beta-selectivity of propranolol, resulting in bronchospasm due to pulmonic beta-2-blockade. The use of a non-selective lipophilic beta-blocker results in several other reported side effects. Restless sleep is probably a direct result of the lipophilic character of propranolol, which allows it to cross the blood brain barrier. A solution to many of the side effects of propranolol therapy may be the use of more selective β-1 antagonists such as metoprolol, which, at low dosage, have little β-2 activity; thus, in theory they bear a lower risk of inducing hypoglycemia and bronchospasm. Treatment with a hydrophilic beta-1 antagonist such as atenolol may prevent side effects, such as restless sleep. However, it is not yet known if these selective beta-blockers will have efficacy that is equal to propranolol. Our study confirms the impressive results of propranolol as a treatment for IH. It seems to be a more effective and safer therapeutic drug than systemic corticosteroids. Its use may be expanded to treatment of IH after the first year of life. Because of potentially harmful side effects, including hypoglycemia, bronchospasm, and hypotension, these patients is preferably treated in a multidisciplinary setting by physicians knowledgeable about the effects and side-effects of propranolol.

### Recurrence

As has been mentioned in several articles, we confirmed that IH successfully treated with propranolol may recur 0–6 months after therapy withdrawal. The rate of clinically visible recurrence of IH in our series of 64 patients was 6 %, suggesting that four cases propranolol does not result in permanent shrinkage. The frequency of recurrences in patients treated with propranolol has not been well-characterized. In the series of cases reported by Sans et al. [9], 2 of 25 patients (8 %) had recurrences after treatment withdrawal. The overall response to retreatment with propranolol was satisfactory. These relapses occurred before the age of 11 months, which might mean that the treatment was withdrawn before the proliferative phase of the IH was over [9]. The cause of relapse in our cases is unknown, and the low number of patients in our series does not allow for definite conclusions. Propranolol withdrawal was considered because the IH had completely stabilized or was almost completely gone. Clinically overt recurrence may indicate that the proliferative phase of the IH was still ongoing, as has been described in hemangiomas with a deep component and segmental morphologic characteristics [27]. In our experience, these two features were more frequent in the recurring group. It is difficult to predict which IH will have a late proliferative phase. The mean duration of treatment in the four recurrent IH in our series was longer than in the non-recurrent cases. Thus, we speculate that it is difficult to establish fixed periods of treatment for patients with IH, and the duration of treatment with propranolol must be individualized according to intrinsic features of the IH or its clinical evolution, which are largely unpredictable. Although no data are available regarding recurrence rate after retreatment withdrawal, in our experience, response to a second treatment with propranolol in the recurrent cases has been satisfactory. In any case, it should be kept in mind that late relapses may occur and that patients with IH treated with propranolol should be followed up for at least 6 months after stopping this treatment. Hemangioma recurrence is also often seen in patients treated with corticosteroids. Although a comparison of the two treatments is not possible, it is the authors’ impression that the rate of recurrence is lower with propranolol than with corticosteroids. The main proposed mechanisms involved in the effectiveness of propranolol for IH include vasoconstriction, inhibition of angiogenesis, inhibition of the renin–angiotensin system, and induction of apoptosis [28, 29]. Apoptosis of endothelial cells in the hemangioma is supposed to be the most likely mechanism involved in its natural involution, and propranolol has been proved to induce apoptosis of such hemangioma cells. As suggested, apoptosis may not be complete in all cases after treatment withdrawal, and thus some endothelial cells may remain proliferative after treatment is stopped [9]. It is unknown why other IH, showing only partial response to propranolol, do not experience further proliferation of the remaining endothelial cells after propranolol withdrawal. The exact mechanism underlying late proliferation of endothelial cells remains unknown. VEGF expression is clearly involved in the proliferative phase of hemangiomas. Lamy demonstrated that propranolol inhibited endothelial proliferation not only through beta-adrenergic receptors, but also by antagonizing the VEGF receptors. Thus, it might be hypothesized that this receptor plays an important role in the recurrence of hemangiomas after propranolol because of the cessation of this inhibitory effect [29].

In conclusion, propranolol has a well-documented safety and mild side effect for infantile hemangiomas. However, propranolol for hemangiomas is an off-label-indication and the parents have to be well informed and to assent. Although we observed a recurrence rate of 6 % of cases of IH treated with propranolol after withdrawal. In all, propranolol appears to be an effective treatment for infantile hamangiomas and should now be used as a first-line treatment in hemangiomas when intervention is required. Also, further studies should be needed in determining the most effective treatment dosage, optimum treatment duration, and exact mechanism of action of propranolol in future.
